# The incidence, risk factors and outcomes of acute kidney injury in critically ill patients undergoing emergency surgery: a prospective observational study

**DOI:** 10.1186/s12882-022-02675-0

**Published:** 2022-01-22

**Authors:** Linhui Hu, Lu Gao, Danqing Zhang, Yating Hou, Lin Ling He, Huidan Zhang, Yufan Liang, Jing Xu, Chunbo Chen

**Affiliations:** 1 Department of Critical Care Medicine, Maoming People’s Hospital, 101 Weimin Road, Maoming, 525000 Guangdong China; 2 Department of Clinical Research Center, Maoming People’s Hospital, 101 Weimin Road, Maoming, 525000 Guangdong China; 3 Intensive Care Unit, The First Affiliated Hospital of Jinan University, Guangzhou, 510630 Guangdong China; 4 Department of Intensive Care Unit, The First Affiliated Hospital of Shantou University Medical College, Shantou, 515041 Guangdong China; 5 Department of Oncology, Maoming People’s Hospital, 101 Weimin Road, Maoming, 525000 Guangdong China; 6 Department of Critical Care Medicine, Guangdong Provincial People’s Hospital, Guangdong Academy of Medical Sciences, 106 Zhongshan Er Road, Guangzhou, 510080 Guangdong China; 7 Department of Intensive Care Unit of Cardiac Surgery, Guangdong Cardiovascular Institute, Guangdong Provincial People’s Hospital, Guangdong Academy of Medical Sciences, 96 Dongchuan Road, Guangzhou, 510080 Guangdong China; 8 The Second School of Clinical Medicine, Southern Medical University, 253 Gongye Dadao Middle, Guangzhou, 510280 China

**Keywords:** Acute kidney injury, Emergency surgery, Critically ill patients, Risk factor, Prognosis

## Abstract

**Background:**

Without sufficient evidence in postoperative acute kidney injury (AKI) in critically ill patients undergoing emergency surgery, it is meaningful to explore the incidence, risk factors, and prognosis of postoperative AKI.

**Methods:**

A prospective observational study was conducted in the general intensive care units (ICUs) from January 2014 to March 2018. Variables about preoperation, intraoperation and postoperation were collected. AKI was diagnosed using the Kidney Disease: Improving Global Outcomes criteria.

**Results:**

Among 383 critically ill patients undergoing emergency surgery, 151 (39.4%) patients developed postoperative AKI. Postoperative reoperation, postoperative Acute Physiology and Chronic Health Evaluation (APACHE II) score, and postoperative serum lactic acid (LAC) were independent risk factors for postoperative AKI, with the adjusted odds ratio (ORadj) of 1.854 (95% confidence interval [CI], 1.091–3.152), 1.059 (95%CI, 1.018–1.102), and 1.239 (95%CI, 1.047–1.467), respectively. Compared with the non-AKI group, duration of mechanical ventilation, renal replacement therapy, ICU and hospital mortality, ICU and hospital length of stay, total ICU and hospital costs were higher in the AKI group.

**Conclusions:**

Postoperative reoperation, postoperative APACHE II score, and postoperative LAC were independent risk factors of postoperative AKI in critically ill patients undergoing emergency surgery.

**Supplementary Information:**

The online version contains supplementary material available at 10.1186/s12882-022-02675-0.

## Introduction

Acute kidney injury (AKI) is a common postoperative complication, with the incidence ranged from 0.8 to 39% described by previous studies [1–3]. The incidence of postoperative AKI ranges varies considerably, which might be related to the diagnostic criteria of AKI and the type of surgery [4]. Studies had shown that the incidence of AKI in critically ill patients was between 31.6 and 67% [5–7]. We speculate that the incidence of postoperative AKI will be high in critically ill patients who have undergone emergency surgery. In contrast, the incidence and risk factors of AKI after emergency surgery in critically ill patients have not been well described.

Postoperative AKI could be potentially fatal, which was mainly manifested by increased hospital mortality [8], prolonged hospital stays, the occurrence of chronic kidney disease (CKD) [9, 10], and accelerated progression to end-stage renal disease (ESRD) [11, 12]. Embedding routinely available data, simple accurate risk scores could be used to predict prognosis [13, 14]. Therefore, it is always a hot topic to clarify the clinical characteristics of postoperative AKI and take effective measures for corresponding prevention and intervention, which is of great clinical significance for improving the safety of patients during the perioperative period. But, for now, most postoperative AKI research focuses on cardiac surgery [15], non-cardiac surgery [16, 17], or neurosurgery [18, 19] at present. Meanwhile, most of the risk factors related to postoperative acute kidney injury are concentrated in specialized operations, so the operation population and operation type are single, which can not reflect the heterogeneities. Hence, research on the incidence, risk factors, and prognosis of postoperative AKI in critically ill patients undergoing emergency surgery was scarce. And it might result in an undesired postponement in initial therapies. A greater understanding of morbidity and risk factors of postoperative AKI after emergency surgery might advance timely diagnosis and treatment. Consequently, we directed this study in adult intensive care units (ICUs) to explore the incidence of postoperative AKI after emergency surgery, recognize perioperative risk factors, elucidate the relationship between postoperative AKI and prognoses, clarify the epidemiological status, and advance the early identification and diagnosis of postoperative AKI.

## Methods

### Study design and participants

This prospective observational research was managed in the general ICUs from Guangdong Provincial People’s Hospital. From January 2014 to March 2018, patients who were admitted to ICU immediately after undergoing noncardiovascular emergency surgery were included. Some patients were already in the ICU prior to the surgery, and some were transferred to the ICU after surgery. Those excluded patients conformed to the criteria that were younger than 18 years, refusal of consent, preexisting ESRD, presence of AKI before emergency surgery, or missing admission data. The primary outcome was defined as the occurrence of AKI according to the Kidney Disease: Improving Global Outcomes (KDIGO) criteria within 1 week after noncardiovascular emergency surgery. And the secondary outcome comprised postoperative duration of mechanical ventilation, postoperative reintubation, postoperative RRT during ICU stay, ICU and hospital mortality, length of ICU and hospital stay, as well as ICU and hospital costs. Following Strengthening the Reporting of Observational Studies in Epidemiology guidelines [20], written informed consent was offered to patients or surrogates for patients’ inability to consent. This research was authorized by the Ethics Committee and executed complying with the Declaration of Helsinki.

### Data collection

Clinical and demographic characteristics and outcomes of these patients were collected once they were admitted to the ICU. Age, gender, body mass index (BMI), preexisting clinical conditions [hypertension, diabetes mellitus, CKD, cerebrovascular disease, and coronary artery disease (CAD)], American Society of Anesthesiologist (ASA) classification, classification of New York Heart Association (NYHA) heart function, preoperative medication including the preoperative use of nephrotoxic drugs [nonsteroidal anti-inflammatory drug (NSAID), angiotensin-converting enzyme inhibitor (ACEI), angiotensin receptor blocker (ARB), immunosuppressant, aminoglycoside, vancomycin, acyclovir, or amphotericin] and the preoperative administration of radiographic contrast, surgery group (neurosurgical surgery, noncardiovascular chest surgery, abdominal surgery, or others), and incision type were registered. Comprising the level of preoperative hemoglobin, baseline serum creatinine (sCr), baseline estimated glomerular filtration rate (eGFR), and concentration of postoperative sCr, hemoglobin, and the lactic acid (LAC) at ICU admission, laboratory data were recorded. Serum creatinine and hemoglobin were detected both preoperation and at least once a day as a part of routine clinical care during ICU hospitalization. The hourly urine output (U.O.) of each patient was also recorded from enrollment to ICU discharge. The postoperative Acute Physiology and Chronic Health Evaluation (APACHE II) score, which was utilized to estimate the patient’s overall condition, was evaluated instantly after anesthesia recovery. Postoperative reoperation within 1 week after the first noncardiovascular emergency surgery was taken notes. Surgical data containing general anesthesia, duration of surgery, intraoperative estimated blood loss, lowest mean arterial pressure (MAP; i.e., lowest MAP for at least five continuous minutes) during anesthesia, radiographic contrast, intraoperative U.O., amount and type of intraoperative fluids administered (crystalloid and artificial colloid), intraoperative transfusions [red blood cells (RBCs), platelets, and plasma] were recorded. Prognosis variables were also recorded, comprising duration of postoperative mechanical ventilation, the incidence of postoperative tracheal reintubation and RRT, ICU and in-hospital mortality, length of stay in hospital and ICU, and total ICU and in-hospital costs.

### Definitions

AKI was diagnosed according to the KDIGO criteria [21] within 1 week after surgery. However, because U.O. criteria could be affected by administrating diuretics or obesity, we adopted serum creatinine to diagnose AKI. The eGFR was calculated using the Chronic Kidney Disease Epidemiology Collaboration (CKD-EPI) creatinine equation [22]. CKD was defined as baseline eGFR < 60 ml/minute/1.73 m2. The baseline sCr was defined as following rules in sequence as described in previous study: (1) the most recent pre-ICU value (between 30 and 365 days before ICU admission); (2) for patients aged < 40 years, a stable pre-ICU value > 365 days before ICU admission (stable defined as within 15% of the lowest ICU measurement); (3) pre-ICU value (> 365 days before ICU admission) and less than the initial sCr at ICU admission; (4) a pre-ICU value (between 3 and 39 days before ICU admission) ≤ initial sCr at the time of admission to ICU and not distinctly in AKI; if patients did not have serum creatine value before ICU admission, (5) the lowest sCr upon initial admission value, the final ICU value, or the minimum value at follow-up unto 365 days [23–25]. Surgical incision was classified into three types, including clean wound (type I), relative clean wounds (type II) and contaminated wounds (type III).

### Sample measurements

All samples were collected simultaneously within 1 h after ICU admission and analyzed at the central laboratory of the Guangdong Provincial People’s Hospital utilizing a standard protocol. The concentrations of samples were measured using commercially available multiplex assays and enzyme-linked immunosorbent assays following the manufacturer’s instructions.

### Statistical analysis

To estimate the multivariable regression coefficients, events per variable (EPV) > 10 was a significant problem [26]. EPV = 10 should be obligatory in this outcome model to avoid bias. Therefore, to meet with a model with 5 covariates, we needed to involve nearly 50 outcome events. With an approximated postoperative AKI incidence of 15%, which was found by previous studies that the incidence of AKI fluctuated from 0.8 to 39% due to different surgical types, we beforehand computed the sample size. Thus, a sample size of 334 cases was essential. Given a possible dropout rate of 10%, we should require at least 368 patients.

SPSS version 16.0 software program (SPSS Inc., Chicago, Illinois, USA) was used in the statistical analyses. A two-sided *P*-value of less than 0.05 was deemed as statistically significant. Mean ± standard deviation (S.D.), median and interquartile range (IQR) performed in continuous variables, while percentages were utilized to present categorical variables. In terms of continuous variables, a *t-*test was used to compare normally distributed variables. At the same time, the Wilcoxon rank-sum test was utilized in the comparison of non-normally distributed variables. Meanwhile, the chi-square test or Fisher’s exact test were used in the comparison of categorical variables. Univariate logistic regression analysis was performed to examine the relationship between each indicator and postoperative AKI separately. We also conducted multivariate logistic regression to evaluate the variables which were independently related to postoperative AKI. A criterion of *P* <  0.10 in the univariate analysis entered into multivariate analysis. Multivariate logistic forward stepwise regression was subsequently utilized to evaluate the most competent predictors of postoperative AKI. OR with 95% confidence intervals (C.I.s) was used to describe the results.

## Results

### Preoperative baseline characteristics of the patients

**Fig. 1:**
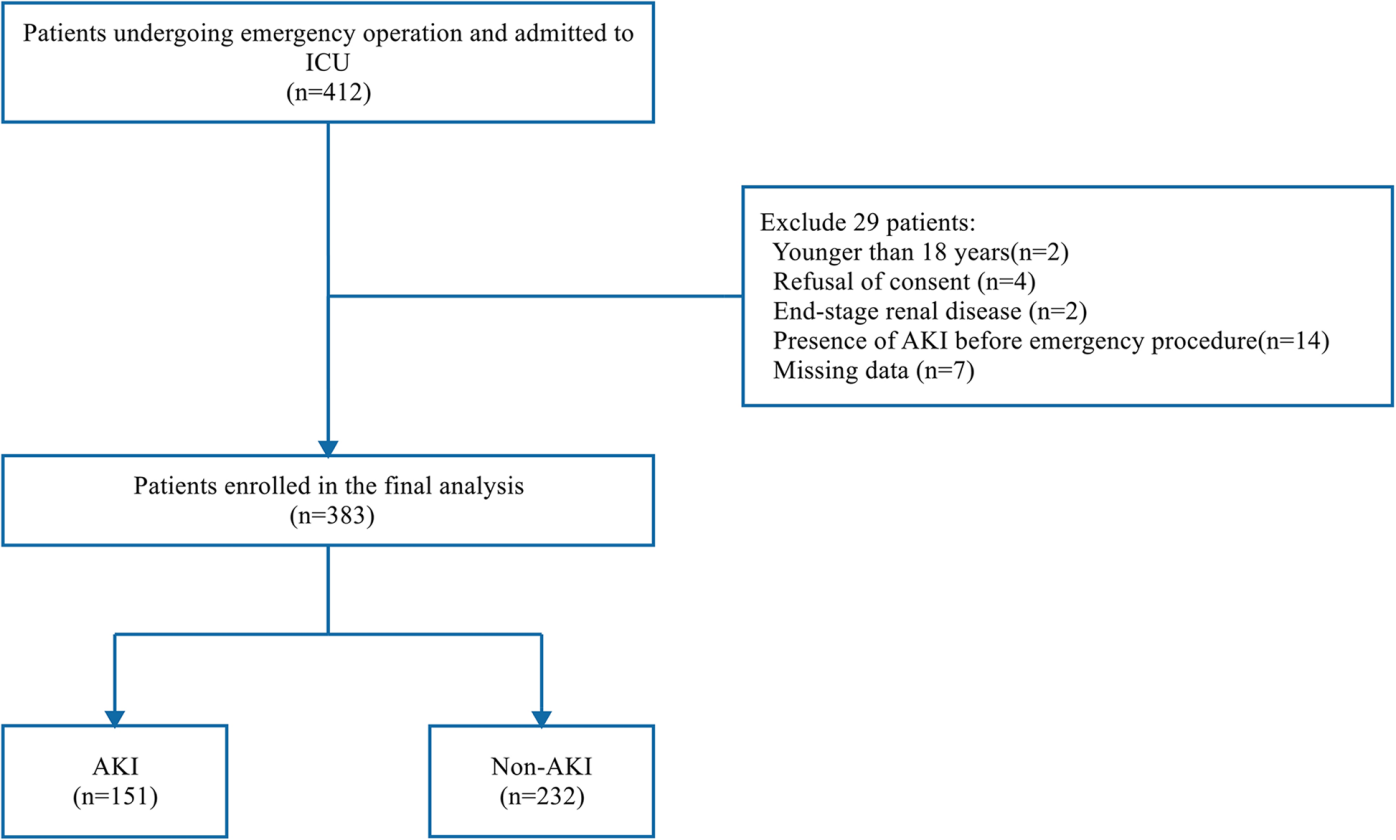
Flow chart from recruitment to the outcome. Abbreviations: ICU, intensive care unit; AKI, acute kidney injury

Figure 1 presented the protocol and flow diagram of the screening process. Among 412 patients enrolled for the study, 29 were excluded due to the subsequent reasons: younger than 18 years (*n* = 2), refusal of consent (*n* = 4), end-stage renal disease (*n* = 2), presence of AKI before emergency procedure (*n* = 14), and missing data (*n* = 7). Finally, a total of 383 patients were involved, among whom 67 (17.5%) were already in the ICU before surgery, and 316 (82.5) were admitted to the ICU after surgery. Of them, 151 (39.4%) patients occurred in postoperative AKI basing on the KDIGO criteria. Of those patients who evolved into postoperative AKI, 92 patients (60.9%) developed to stage 1, 40 patients (26.5%) were progressed to stage 2, and 19 patients were evolved into stage 3 (12.6%). Among the 151 postoperative AKI patients, 110 (72.8%) developed postoperative AKI on the first day after the operation, 25 (16.6%) on the second day, 6 (4.0%) on the third day, and 10 (6.6%) patients beyond 3 days. Thus, 93.4% of the patients reached postoperative AKI within 3 days after emergency operations.

As presented in Table 1, compared with non-AKI patients, patients with AKI had a significantly higher rate of preexisting clinical conditions of hypertension, and a larger proportion of patients in the AKI group underwent abdominal surgery than in the non AKI group. Moreover, both ASA classification and Classification of NYHA heart function were significantly higher in patients involving in postoperative AKI. Whereas, compared with postoperative AKI, there were no significant differences in age, sex, BMI, the preexisting clinical conditions (including diabetes mellitus, cerebrovascular disease, CAD), preoperative hemoglobin concentration, baseline sCr, baseline eGFR, preoperative medication of radiographic contrast, some varieties of surgery group (containing noncardiovascular chest surgery, others) and incision type in patients without postoperative AKI. Contrary to expectations, there was no marked difference between postoperative AKI patients and non-postoperative-AKI patients in the preoperative medication of nephrotoxic drugs.

**Table 1:** Preoperative baseline characteristics of the patients

Characteristics	All patients (***n*** = 383)	Non-AKI (***n*** = 232)	AKI (***n*** = 151)	***P*** value
Age, years	60(49,71)	59(49,71)	61(49,71)	0.996
Gender (male), n (%)	247(64.5)	149(64.2)	98(64.9)	0.892
BMI, kg/m2	23.9(22.1,25.5)	23.8(22.1,25.4)	24.1(22.0,25.6)	0.173
Preexisting clinical conditions, n (%)				
Hypertension	161(42.0)	86(37.1)	75(49.7)	0.015
Diabetes mellitus	37(9.7)	17(7.3)	20(13.2)	0.055
Cerebrovascular disease	157(41.0)	103(44.4)	54(35.8)	0.093
CAD	35(9.1)	18(7.8)	17(11.3)	0.245
ASA classification, n (%)				0.002
I	31(8.1)	19(8.2)	12(7.9)	
II	139(36.3)	102(44.0)	38(24.5)	
III	154(40.2)	86(37.1)	68(45.0)	
IV	49(12.8)	23(9.9)	26(17.2)	
V	5(1.3)	1(0.4)	4(2.6)	
Classification of NYHA heart function				0.015
I	175(45.7)	115(49.6)	60(39.7)	
II	181(47.3)	107(46.1)	74(49.0)	
III	27(7.0)	10(4.3)	17(11.3)	
Preoperative hemoglobin, g/L	123.0(99.3138.5)	123.8(105.2139.6)	121.0(92.0,135.5)	0.063
Baseline serum creatinine, mg/dl	0.78(0.62,1.01)	0.79(0.64,0.99)	0.72(0.59,1.07)	0.373
Baseline eGFR, ml/min/1.73 m^2^	94.75(72.79,107.95)	94.0(75.4106.8)	95.3(68.2110.3)	0.852
Preoperative medication, n (%)				
Nephrotoxic drugs	37(9.7)	17(7.3)	20(13.2)	0.055
Radiographic contrast	100(26.1)	63(27.2)	37(24.5)	0.564
Surgery group, n (%)				
Neurosurgical surgery	187(48.8)	124(53.4)	63(41.7)	0.025
Noncardiovascular chest surgery	6(1.6)	4(1.7)	2(1.3)	1.000
Abdominal surgery	161(42.0)	82(35.3)	79(52.3)	0.001
Others	29(7.6)	22(9.5)	7(4.6)	0.080
Incision type				0.614
I	258(67.4)	160(69.0)	98(64.9)	
II	104(27.2)	61(26.3)	43(28.5)	
III	21(5.5)	11(4.7)	10(6.6)	

The continuous variables were expressed as mean ± SD or median (25th percentile–75th percentile, IQR). Categorical variables were expressed as a number (%). Nephrotoxic drugs include any of the following medications administered within 5 days before the operation: nonsteroidal anti-inflammatory drug, angiotensin-converting enzyme inhibitor, angiotensin receptor blocker, immunosuppressant, aminoglycoside, vancomycin, acyclovir, amphotericin

Abbreviations: *AKI* Acute Kidney Injury, *ASA classification* American Society of Anesthesiologists Classification, *BMI* Body Mass Index, *CAD* Coronary Artery Disease, *eGFR* Estimated Glomerular Filtration Rate, *IQR* Interquartile Range, *NYHA* New York Heart Association, *SD* Standard Deviation

### Intraoperative characteristics of the patients

Table 2 demonstrated the intraoperative parameters of patients in this cohort. During the operation, patients who developed postoperative AKI were accompanied by a longer duration of surgery, more estimated blood loss, and lower minimum MAP. Meanwhile, postoperative AKI patients had a higher rate of radiographic contrast. Additionally, the patients who accepted total artificial colloid or blood transfusion (RBCs, plasma, platelets) during the operation were more likely to develop postoperative AKI. However, the general anesthesia, intraoperative U.O., and total crystalloid of intraoperative fluids were inconspicuous for postoperative AKI.

**Table 2:** Intraoperative characteristics of the patients

Variables	All patients (***n*** = 383)	Non-AKI (***n*** = 232)	AKI (***n*** = 151)	***P*** value
General anesthesia, n (%)	370 (96.6)	224 (96.6)	146 (96.7)	0.942
Duration of surgery, minute	196 (141,270)	189 (131,256)	200 (151,295)	0.015
Estimated blood loss,ml	50.0 (5.0,250.0)	50.0 (5.0,200.0)	100.0 (10.0,400.0)	0.003
Minimum MAP, mm Hg	75.0 (67.0,80.0)	77.0 (68.5,81.0)	73.0 (63.0,80.0)	0.003
Radiographic contrast, n (%)	92 (24.0)	64 (27.6)	28 (18.5)	0.043
Intraoperative UO, ml/kg/h	1.15 (0.41,2.64)	1.28 (0.23,2.82)	0.98 (0.47,2.18)	0.235
Intraoperative fluids				
Total crystalloid, per 1000 ml	0.5 (0.5,1.0)	0.5 (0.5,1.0)	0.5 (0.5,1.5)	0.285
Total artificial colloid, per 1000 ml	1.0 (0.5,1.0)	1.0 (0.5,1.0)	1.0 (0.5,1.5)	0.020
RBCs, n (%)	121 (31.6)	55 (23.7)	66 (43.7)	< 0.0001
Plasma, n (%)	102 (26.6)	45 (19.4)	57 (37.7)	< 0.0001
Platelets, n (%)	13 (3.4)	4 (1.7)	9 (6.0)	0.025

Continuous variables were presented as mean ± SD or median (25th percentile–75th percentile, IQR). Categorical variables were expressed as a number (%)

Abbreviations: *AKI* Acute Kidney Injury, *MAP* Mean Arterial Pressure, *IQR* Interquartile Range, *RBC* Red Bood Cell, *SD* Standard Deviation, *UO* Urine Output

### Postoperative characteristics of the patients

Revealing by Table 3, patients with more potential to develop postoperative AKI had the following features: the higher APACHE II score reflected the severity of the disease and the patient’s overall situation, the higher concentration of the postoperative sCr and postoperative LAC concentration. In addition, patients who went through postoperative AKI had a greater likelihood of developing oliguria and need for undergoing reoperation after the first emergency surgery.

**Table 3:** Postoperative characteristics of the patients

Variables	All patients (***n*** = 383)	Non-AKI (***n*** = 232)	AKI (***n*** = 151)	***P*** value
APACHE II score	18(13,22)	16(12,21)	20(16,25)	< 0.0001
Serum Cr, mg/dl	0.96(0.74,1.37)	0.85(0.69,1.09)	1.28(0.89,1.86)	< 0.0001
Hemoglobin, g/L	110.0(95.0,121.6)	112.7(97.0,122.7)	107.0(89.0,121.0)	0.086
Lactic acid,mmol/L	1.5(0.9,2.4)	1.2(0.8,2.1)	1.8(1.1,3.3)	< 0.0001
UP, ml/kg/h	1.79(1.17,2.41)	1.85(1.24,2.45)	1.58(1.04,2.38)	0.033
Postoperative reoperation, n (%)	97(25.3)	52(22.4)	45(29.8)	0.048

Continuous variables were expressed as mean ± SD or median (25th percentile–75th percentile, IQR). Categorical variables were expressed as a number (%). Postoperative reoperation, need for the second emergency operation within 7 days after the first emergency procedure

Abbreviations: *AKI* Acute Kidney Injury, *APACHE II* Acute Physiology and Chronic Health Evaluation II, *Cr* Creatinine, *IQR* Interquartile Range, *UP* Urine Output Within The First 24 Hours After Operation, *SD* Standard Deviation

### Univariable and multivariable analysis of risk factors that are associated with postoperative AKI

Univariate analysis showed that hypertension, ASA classification, classification of NYHA heart function, preoperative hemoglobin, neurosurgical surgery, abdominal surgery, duration of surgery, estimated blood loss, minimum MAP, radiographic contrast, intraoperative U.O., total infused artificial colloid, RBCs or plasma, postoperative APACHE II score, postoperative sCr, postoperative hemoglobin, postoperative U.O., postoperative lactic acid, and postoperative reoperation were risk factors that are associated with postoperative AKI (Table S1). Since postoperative sCr and U.O. are a manifestation and a diagnostic criterion for AKI, postoperative sCr and U.O. were not included in the multivariate study to analyze whether it was a risk factor for AKI. As for postoperative AKI, postoperative reoperation, postoperative APACHE II score, and postoperative LAC concentration were the independent risk factors after multivariable adjustment, which was shown in Table 4. Postoperative reoperation was an independent risk factor of postoperative AKI with the adjusted OR (ORadj) of 1.854 (95% CI, 1.091–3.152). At the same time, we discovered that higher postoperative APACHE II score and postoperative LAC could be used to predict postoperative AKI incidence with [ORadj 1.059 (95% CI, 1.018–1.102)] and [ORadj 1.239 (95% CI, 1.047–1.467)], respectively.

**Table 4:** Multivariable logistic regression analysis of factors that are related to postoperative AKI in emergency operation for critically ill patients

Variable	ORunadj	ORadj	95% CI	***P*** value
Postoperative reoperation	1.595	1.854	1.091–3.152	0.022
Postoperative APACHE II score	1.098	1.059	1.018–1.102	0.005
Postoperative lactic acid,mmol/L	1.392	1.239	1.047–1.467	0.013

Independent variables including ASA classification, classification of NYHA heart function, preoperative hemoglobin, neurosurgical surgery, abdominal surgery, duration of surgery, estimated blood loss, minimum MAP, radiographic contrast, intraoperative UO, total artificial colloid, RBCs, plasma, postoperative APACHE II score, postoperative hemoglobin, postoperative lactic acid, and postoperative reoperation were involved in the stepwise analysis. Postoperative reoperation, need for the second emergency operation within 7 days after the first emergency procedure

Abbreviations: *AKI* Acute Kidney Injury, *ASA classification* American Society of Anesthesiologists Classification, *NYHA* New York Heart Association, *MAP* Mean Arterial Pressure, *UO* Urine Output; RBC, Red Bood Cell, *APACHE II* Acute Physiology and Chronic Health Evaluation, *CI* Confidence Interval, *ORadj* Odds Ratio Adjusted, *ORunadj* Odds Ratio Without Adjusted

### Clinical outcomes of postoperative patients

Elucidated in Table 5, the occurrence of postoperative AKI would lead to higher rates of postoperative RRT, ICU mortality, and in-hospital mortality. Moreover, patients with postoperative AKI were more likely to go through the long duration of postoperative mechanical ventilation, prolonging ICU and hospital length of stay, higher total ICU costs, and higher hospital ICU costs. But there was no marked difference between postoperative AKI and reintubation.

**Table 5:** Clinical outcomes of postoperative patients

Outcomes	All patients (***n*** = 383)	Non-AKI (***n*** = 232)	AKI (***n*** = 151)	***P*** value
Duration of mechanical Ventilation, hours	12(4,68)	9(2,22)	36(10,145)	< 0.001
Reintubation, n (%)	44(11.5)	21(9.1)	23(15.2)	0.064
RRT, n (%)	21(5.5)	3(1.3)^a^	18(11.9)	< 0.001
ICU mortality, n (%)	72(18.8)	31(13.4)	41(27.2)	0.001
Hospital mortality, n (%)	77(20.1)	33(14.2)	44(29.1)	< 0.001
ICU length of stay, days	4(2,9)	2(1,6)	8(4,15)	< 0.001
Hospital length of stay, days	14(9,25)	12(8,19)	21(12,34)	< 0.001
Total ICU cost, CNY	34,038(14,132,77,231)	20,081(11,415,40,776)	60,909(34,338,126,413)	< 0.001
Total Hospital cost, CNY	98,793(59,651,173,582)	80,639(50,722,126,803)	151,384(87,973,223,422)	< 0.001

^a^There are 3 patients who had AKI 7 days after surgery need to receive renal replacement therapy (RRT). Continuous variables were expressed as mean ± SD or median (25th percentile–75th percentile, IQR); Categorical variables were expressed as a number (%)

Abbreviations: *AKI* Acute Kidney Injury, *CNY* Chinese Yuan, *ICU* Intensive Care Unit, *IQR* Interquartile Range, *RRT* Renal Replacement Therapy, *SD* Standard Deviation

## Discussion

In this prospective study, we found that the morbidity of postoperative AKI was as high as 39.40% in critically ill patients undergoing emergency surgery, and the occurrence of postoperative AKI would further lead to adverse hospitalization results. Compared with previous studies, there was distinction for the morbidity of postoperative AKI, while the high risk of negative hospitalization results was consistent [27, 28]. Considering the heterogeneousness of the population in critically ill patients undergoing emergency surgery and the numerous kinds of operations, the incidence of postoperative AKI found in our research was higher than in previous studies. Furthermore, our study found that 93.40% of the patients come through postoperative AKI within 3 days after emergency operations, so physicians must take early surveillance and early intervention for those at high risk of postoperative AKI. Therefore, with a large sample size, high population heterogeneity, and a wide range of surgeries, our research results have strong applicability and popularization in clinical practice.

The independent risk factors of postoperative AKI incidence included postoperative reoperation, postoperative APACHE II score, and postoperative LAC. Unlike previous Meta-analyses, our study found that BMI, postoperative mechanical ventilation duration, and other factors were not risk factors for AKI [29]. The risk factors for postoperative AKI varied in different clinical situations, and three of the above risk factors were recognized in this emergency surgery cohort. It was previously reported that reoperation was identified as one of the independent predictors of AKI in patients undergoing cardiac surgery, abdominal surgery and neurosurgery [18, 30, 31]. Manifested by this study, postoperative reoperation was an independent risk factor for the occurrence of postoperative AKI, which was consistent with previous studies. However, in our study, 93.40% of the patients was diagnosed with AKI within 3 days after emergency operations, the possible reason for which might be the poor overall clinical conditions of patients who need secondary operation, which leads the continuous elevation of the serum creatinine. Although the mechanism of reoperation leading to postoperative AKI has not been fully elucidated, it is theoretically believed that factors such as hemodynamic damage, bleeding, and poor overall condition involved in reoperation are related to postoperative AKI. Under the action of these factors, the body is more likely to cause overexcitation of the sympathetic-adrenal medullary system, promote the increase in plasma catecholamine concentration, cause neurohumoral regulation dysfunction, and lead to the degeneration and necrosis of epithelial cells due to ischemia and hypoxia, and finally the occurrence of postoperative AKI [32].

The APACHE II scoring system was usually utilized to evaluate the severity and prognosis of widespread diseases, which could more objectively reflect and comprehensively assess the current pathophysiology of patients [33]. The higher APACHE II score indicates the more severity of the patient’s overall condition and the greater risk of death [34, 35], thus the patient is more susceptible to postoperative AKI. As expected, the postoperative APACHE II score was closely associated with AKI in this study, which was in consistent with the previous study in postoperative cohort [36]. Since there are many variables involved in the APACHE II scoring system, which including the vital signs, oxygenation state, electrolyte levels, sCr, blood routine examination and the consciousness state, any change in one item may lead to a different result. Therefore, improving the postoperative physical state might help reduce the morbidity of postoperative AKI. However, the inclusion of creatinine into the APACHE II score might have a significant influence on the association between APACHE II with AKI.

It was previously reported that lactic acid was related to AKI occurrence in military casualties, trauma patients, and patients undergoing liver transplantation [37–39]. Nevertheless, the similar conclusion was not shown in the meta-analysis of observational studies conducted by Cartin-Ceba R.et al. [40]. In our study, the LAC level in the postoperative AKI group was significantly increased. The elevation of postoperative serum LAC concentration is associated with perioperative hypoxia and hypoperfusion, which causes an increase in catecholamine, resulting in accelerated glycolysis, the release of systemic inflammatory mediators, and decreased liver and kidney clearance [41]. Therefore, the elevated LAC level would be a predictor of postoperative AKI occurrence.

More and more studies had shown that even relatively mild renal injury drugs were related to increased risk of AKI morbidity and mortality [13]. In this study, we also analyzed commonly prescribed medications that predispose to renal impairment, including NSAID, ACEI, ARB, immunosuppressants, aminoglycosides, vancomycin, acyclovir, and amphotericin. Even though nephrotoxic drugs were well known for their kidney damage, their use had little to do with the occurrence of AKI in this study. We could not obtain statistically significant conclusions due to the small number of patients utilizing these drugs in our cohort. Simultaneously, physicians in ICU paid more attention to carefully evaluate drugs in their potential injury to renal function and structure to avoid damage in the kidney, which was consistent with the KDIGO standard.

As shown in a national study conducted by Y Sanaiha et al., the increasing incidence of acute kidney injury and renal replacement therapy after an emergency general surgery would lead to greater odds of mortality and greater costs of hospitalization and duration of stay [28]. Therefore, this study aimed to discuss the risk factors and morbidity of postoperative AKI in critically ill patients undergoing emergency surgery to prevent the occurrence of complications, which had crucial clinical significance. Despite the lack of effective treatment options, assessing the risk factors and morbidity of postoperative AKI might help formulate new strategies to prevent postoperative AKI, and provide guidance for the clinical physicians to communicate with patients and their families. It was worth noting that all the above-identified risk factors and morbidity in our study were verifiable. However, before applying our study findings to clinical practice, further intervention studies must be conducted to confirm the effectiveness of these risk factors.

This study is the first prospective observational study of postoperative AKI in critically ill patients undergoing emergency surgery to provide the basis for defining the epidemiological status and improving clinical prevention strategies for AKI in critically ill patients undergoing emergency surgery. However, our study still had some limitations and shortcomings. First, this was a single-center prospective study. Therefore, the influence of some confounding factors could not be completely ruled out, which might further lead to a particular deviation in the judgment of incidence, influencing factors, and prognosis. In addition, it needed to be verified by a large sample, multicenter prospective study to reduce bias. Secondly, the data of this study were collected from the general ICU and did not fully represent all postoperative ICU patients, especially those who were undergoing cardiovascular surgery. Moreover, this study lacked long-term follow-up after discharge and failed to count the kidney’s long-term prognosis.

## Conclusion

The morbidity of postoperative AKI in critically ill patients undergoing emergency surgery according to the KDIGO standard was 39.4, and 93.4% of postoperative AKI occurred within 3 days after emergency operations. Postoperative reoperation, postoperative APACHE II score, and postoperative LAC were independent risk factors of the incidence of postoperative AKI in critically ill patients undergoing emergency surgery. In addition, there was a close relationship between postoperative AKI and adverse hospital prognoses. Therefore, this study had crucial clinical significance for critically ill patients undergoing emergency surgery who were at risk of postoperative AKI.

## Supplementary Information

**Additional file 1: Table S1.** Univariable logistic regression analysis of factors that are related to postoperative AKI in emergency operation for critically ill patients.

## Data Availability

All data generated or analyzed during this study are included in this published article. Additional information about the data is available from the corresponding author on reasonable request.

## References

[1] Zhao BC, Shen P, Liu KX (2017). Perioperative statins do not prevent acute kidney injury after cardiac surgery: a meta-analysis of randomized controlled trials. J Cardiothorac Vasc Anesth.

[2] Yang X, Chen C, Teng S, Fu X, Zha Y, Liu H, Wang L, Tian J, Zhang X, Liu Y (2017). Urinary matrix Metalloproteinase-7 predicts severe AKI and poor outcomes after cardiac surgery. J Am Soc Nephrol.

[3] Maxwell RA, Bell CM (2017). Acute kidney injury in the critically ill. Surg Clin North Am.

[4] Grams ME, Sang Y, Coresh J, Ballew S, Matsushita K, Molnar MZ, Szabo Z, Kalantar-Zadeh K, Kovesdy CP (2016). Acute kidney injury after major surgery: a retrospective analysis of veterans health administration data. Am J Kidney Dis.

[5] Fang M, Liu S, Zhou Y, Deng Y, Yin Q, Hu L, Ouyang X, Hou Y, Chen C (2019). Circular RNA involved in the protective effect of losartan on ischemia and reperfusion induced acute kidney injury in rat model. Am J Transl Res.

[6] Wu Y, Peng W, Wei R, Zhou Y, Fang M, Liu S, Deng Y, Yin Q, Ouyang X, Hu L (2019). Rat mRNA expression profiles associated with inhibition of ischemic acute kidney injury by losartan. Biosci Rep.

[7] Zhang D, Gao L, Ye H, Chi R, Wang L, Hu L, Ouyang X, Hou Y, Deng Y, Long Y (2019). Impact of thyroid function on cystatin C in detecting acute kidney injury: a prospective, observational study. BMC Nephrol.

[8] van den Akker JP, Egal M, Groeneveld AB (2013). Invasive mechanical ventilation as a risk factor for acute kidney injury in the critically ill: a systematic review and meta-analysis. Crit Care.

[9] Schiffl H, Lang SM, Fischer R (2012). Long-term outcomes of survivors of ICU acute kidney injury requiring renal replacement therapy: a 10-year prospective cohort study. Clin Kidney J.

[10] Martins CB, Bels D, Honore PM, Redant S (2020). Early prediction of acute kidney injury by machine learning: should we add the urine output criterion to improve this new tool?. J Transl Int Med.

[11] Chen C, Yang X, Lei Y, Zha Y, Liu H, Ma C, Tian J, Chen P, Yang T, Hou FF (2016). Urinary biomarkers at the time of AKI diagnosis as predictors of progression of AKI among patients with acute Cardiorenal syndrome. Clin J Am Soc Nephrol.

[12] Yang X, Chen C, Tian J, Zha Y, Xiong Y, Sun Z, Chen P, Li J, Yang T, Ma C (2015). Urinary angiotensinogen level predicts AKI in acute decompensated heart failure: a prospective, two-stage study. J Am Soc Nephrol.

[13] Lysak N, Hashemighouchani H, Davoudi A, Pourafshar N, Loftus TJ, Ruppert M, Efron PA, Rashidi P, Bihorac A, Ozrazgat-Baslanti T (2020). Cardiovascular death and progression to end-stage renal disease after major surgery in elderly patients. BJS Open.

[14] Deng Y, Ma J, Hou Y, Zhou D, Hou T, Li J, Liang S, Tan N, Chen C (2020). Combining serum Cystatin C and urinary N-acetyl-Beta-D-Glucosaminidase improves the precision for acute kidney injury diagnosis after resection of intracranial space-occupying lesions. Kidney Blood Press Res.

[15] Liu Y, Li H, Chen S, Chen J, Tan N, Zhou Y, Liu Y, Ye P, Ran P, Duan C (2016). Excessively high hydration volume may not be associated with decreased risk of contrast-induced acute kidney injury after percutaneous coronary intervention in patients with renal insufficiency. J Am Heart Assoc.

[16] Bell S, Dekker FW, Vadiveloo T, Marwick C, Deshmukh H, Donnan PT, Van Diepen M (2015). Risk of postoperative acute kidney injury in patients undergoing orthopaedic surgery--development and validation of a risk score and effect of acute kidney injury on survival: observational cohort study. BMJ.

[17] Bell S, Davey P, Nathwani D, Marwick C, Vadiveloo T, Sneddon J, Patton A, Bennie M, Fleming S, Donnan PT (2014). Risk of AKI with gentamicin as surgical prophylaxis. J Am Soc Nephrol.

[18] Deng Y, Yuan J, Chi R, Ye H, Zhou D, Wang S, Mai C, Nie Z, Wang L, Zhai Y (2017). The incidence, risk factors and outcomes of postoperative acute kidney injury in neurosurgical critically ill patients. Sci Rep.

[19] Kovacheva VP, Aglio LS, Boland TA, Mendu ML, Gibbons FK, Christopher KB (2016). Acute kidney injury after craniotomy is associated with increased mortality: a cohort study. Neurosurgery.

[20] von Elm E, Altman DG, Egger M, Pocock SJ, Gøtzsche PC, Vandenbroucke JP (2014). The strengthening the reporting of observational studies in epidemiology (STROBE) statement: guidelines for reporting observational studies. Int J Surg.

[21] Khwaja A (2012). KDIGO clinical practice guidelines for acute kidney injury. Nephron Clin Pract.

[22] Levey AS, Stevens LA, Schmid CH, Zhang YL, Castro AF, Feldman HI, Kusek JW, Eggers P, Van Lente F, Greene T (2009). A new equation to estimate glomerular filtration rate. Ann Intern Med.

[23] Zarbock A, Kellum JA, Schmidt C, Van Aken H, Wempe C, Pavenstädt H, Boanta A, Gerß J, Meersch M (2016). Effect of early vs delayed initiation of renal replacement therapy on mortality in critically ill patients with acute kidney injury: the ELAIN randomized clinical trial. Jama.

[24] Deng Y, Wang L, Hou Y, Ma J, Chi R, Ye H, Zhai Y, Zhang D, Gao L, Hu L (2019). The influence of glycemic status on the performance of cystatin C for acute kidney injury detection in the critically ill. Ren Fail.

[25] Endre ZH, Walker RJ, Pickering JW, Shaw GM, Frampton CM, Henderson SJ, Hutchison R, Mehrtens JE, Robinson JM, Schollum JB (2010). Early intervention with erythropoietin does not affect the outcome of acute kidney injury (the EARLYARF trial). Kidney Int.

[26] Steyerberg EW, Schemper M, Harrell FE (2011). Logistic regression modeling and the number of events per variable: selection bias dominates. J Clin Epidemiol.

[27] O'Connor ME, Kirwan CJ, Pearse RM, Prowle JR (2016). Incidence and associations of acute kidney injury after major abdominal surgery. Intensive Care Med.

[28] Sanaiha Y, Kavianpour B, Dobaria V, Mardock AL, Rudasill S, Lyons R, Benharash P (2020). Acute kidney injury is independently associated with mortality and resource use after emergency general surgery operations. Surgery.

[29] Trongtrakul K, Sawawiboon C, Wang AY, Chitsomkasem A, Limphunudom P, Kurathong S, Prommool S, Trakarnvanich T, Srisawat N (2019). Acute kidney injury in critically ill surgical patients: epidemiology, risk factors and outcomes. Nephrology (Carlton).

[30] Karkouti K, Wijeysundera DN, Yau TM, Callum JL, Cheng DC, Crowther M, Dupuis JY, Fremes SE, Kent B, Laflamme C (2009). Acute kidney injury after cardiac surgery: focus on modifiable risk factors. Circulation.

[31] Long TE, Helgason D, Helgadottir S, Palsson R, Gudbjartsson T, Sigurdsson GH, Indridason OS, Sigurdsson MI (2016). Acute kidney injury after abdominal surgery: incidence, risk factors, and outcome. Anesth Analg.

[32] Bagshaw SM, George C, Gibney RT, Bellomo R (2008). A multi-center evaluation of early acute kidney injury in critically ill trauma patients. Ren Fail.

[33] Deng Y, Chi R, Chen S, Ye H, Yuan J, Wang L, Zhai Y, Gao L, Zhang D, Hu L (2017). Evaluation of clinically available renal biomarkers in critically ill adults: a prospective multicenter observational study. Crit Care.

[34] Hu B, Lv B, Chen C (2018). The choice of a postpyloric tube and the patient's position in our procedure: a response. Crit Care.

[35] Ma J, Deng Y, Lao H, Ouyang X, Liang S, Wang Y, Yao F, Deng Y, Chen C (2021). A nomogram incorporating functional and tubular damage biomarkers to predict the risk of acute kidney injury for septic patients. BMC Nephrol.

[36] Sang L, Chen S, Nong L, Xu Y, Liang W, Zheng H, Zhou L, Sun H, He J, Liu X (2021). The prevalence, risk factors, and prognosis of acute kidney injury after lung transplantation: a single-center cohort study in China. Transplant Proc.

[37] Heegard KD, Stewart IJ, Cap AP, Sosnov JA, Kwan HK, Glass KR, Morrow BD, Latack W, Henderson AT, Saenz KK (2015). Early acute kidney injury in military casualties. J Trauma Acute Care Surg.

[38] Bihorac A, Delano MJ, Schold JD, Lopez MC, Nathens AB, Maier RV, Layon AJ, Baker HV, Moldawer LL (2010). Incidence, clinical predictors, genomics, and outcome of acute kidney injury among trauma patients. Ann Surg.

[39] Barreto AG, Daher EF, Silva Junior GB, Garcia JH, Magalhães CB, Lima JM, Viana CF, Pereira ED (2015). Risk factors for acute kidney injury and 30-day mortality after liver transplantation. Ann Hepatol.

[40] Cartin-Ceba R, Kashiouris M, Plataki M, Kor DJ, Gajic O, Casey ET (2012). Risk factors for development of acute kidney injury in critically ill patients: a systematic review and meta-analysis of observational studies. Crit Care Res Pract.

[41] Nogi K, Shiraishi A, Yamamoto R, Sasano M, Matsumoto T, Karumai T, Hayashi Y (2019). Intermittent hemodialysis for managing metabolic acidosis during resuscitation of septic shock: a descriptive study. Crit Care Explor.

