# Supplementary material for: The incidence, risk factors and outcomes of acute kidney injury in critically ill patients undergoing emergency surgery: a prospective observational study
Source: BMC Nephrol. 2022 Jan 22;23:42. doi: 10.1186/s12882-022-02675-0 (PMC8782702; doi:10.1186/s12882-022-02675-0)
Supplement: Supplementary file 1 — Additional file 1: Table S1. Univariable logistic regression analysis of factors that are related to postoperative AKI in emergency operation for critically ill patients. [file 12882_2022_2675_MOESM1_ESM.docx]

**Table S1. Univariable logistic regression analysis of factors that are related to postoperative AKI in emergency operation for critically ill patients.**

| **Variable** | **ORunadj** | **95% CI** | ***P* value** |
| --- | --- | --- | --- |
| Hypertension | 1.675 | 1.106-2.539 | 0.015 |
| ASA classification | 1.524 | 1.188-1.953 | 0.001 |
| Classification of NYHA heart function | 1.560 | 1.114-2.183 | 0.010 |
| Preoperative hemoglobin, g/L | 0.992 | 0.984-0.999 | 0.033 |
| Neurosurgical surgery | 0.624 | 0.412-0.943 | 0.025 |
| Abdominal surgery | 2.007 | 1.322-3.048 | 0.001 |
| Duration of surgery, minute | 1.002 | 1.001-1.004 | 0.007 |
| Estimated blood loss, ml | 1.000 | 1.000-1.001 | 0.008 |
| Minimum MAP, mmHg | 0.968 | 0.950-0.986 | 0.001 |
| Radiographic contrast, n (%) | 0.598 | 0.362-0.987 | 0.044 |
| Intraoperative UO, ml/kg/h | 0.902 | 0.814-0.998 | 0.046 |
| Total artificial colloid, per 1000 ml | 1.242 | 1.066-1.448 | 0.005 |
| RBCs, n (%) | 2.499 | 1.607-3.886 | <0.001 |
| Plasma, n (%) | 2.520 | 1.586-4.003 | <0.001 |
| Postoperative APACHE II score | 1.098 | 1.060-1.138 | <0.001 |
| Postoperative sCr, mg/dl | 5.195 | 3.213-8.399 | <0.001 |
| Postoperative hemoglobin, g/L | 0.988 | 0.979-0.997 | 0.011 |
| Postoperative UO, ml/kg/h | 0.815 | 0.666-0.999 | 0.048 |
| Postoperative lactic acid, mmol/L | 1.392 | 1.199-1.615 | <0.001 |
| Postoperative reoperation, n (%) | 1.595 | 1.000-2.544 | 0.049 |

Postoperative reoperation, need for the second emergency operation within 7 days after the first emergency procedure. Abbreviations: AKI, Acute Kidney Injury; ASA classification, American Society of Anesthesiologists Classification; NYHA, New York Heart Association; MAP, Mean Arterial Pressure; UO, Urine Output; RBC, Red Bood Cell; APACHE II, Acute Physiology and Chronic Health Evaluation; sCr, serum creatinine; CI, Confidence Interval; ORunadj, Odds Ratio Without Adjusted.
